# Oncogenic MicroRNAs: Key Players in Malignant Transformation

**DOI:** 10.3390/cancers7040904

**Published:** 2015-12-18

**Authors:** Tania Frixa, Sara Donzelli, Giovanni Blandino

**Affiliations:** Translational Oncogenomics Laboratory, Regina Elena National Cancer Institute, 00144 Rome, Italy

**Keywords:** cancer, oncogenic miRNAs, cell cycle regulation, apoptosis

## Abstract

MicroRNAs (miRNAs) represent a class of non-coding RNAs that exert pivotal roles in the regulation of gene expression at the post-transcriptional level. MiRNAs are involved in many biological processes and slight modulations in their expression have been correlated with the occurrence of different diseases. In particular, alterations in the expression of miRNAs with oncogenic or tumor suppressor functions have been associated with carcinogenesis, malignant transformation, metastasis and response to anticancer treatments. This review will mainly focus on oncogenic miRNAs whose aberrant expression leads to malignancy.

## 1. Introduction 

Alterations in DNA, caused by genetic mutations determining structural and expression modification, at coding and non-coding regions, are responsible for cancer occurrence, gradual increase in tumor size, disorganization and malignancy [1]. Coding regions refer to oncogenes and tumor-suppressor genes whose alterations have been thought for a long time to be the only causes of tumorigenesis. These genes are involved in the main biological pathways, thus their control is the key of the cellular homeostasis [2]. Recent studies have widely discussed of a large fraction of non-coding RNA (ncRNA) transcripts with no significant open reading frame, which are involved in many biological processes such as cell cycle control, apoptosis, development and differentiation, and result to be aberrantly expressed in tumors [3]. Some ncRNA are called microRNAs (miRNAs), which are small non-coding RNAs of approximately 20–22 nucleotides in length in their mature form. 

The process by which a precursor miRNA becomes a mature miRNA involves different biological steps. [4,5]. The biogenesis of miRNAs starts in the nucleus with the transcription of the primary miRNA (pri-miRNA) that undergoes many steps of maturation. The maturation process begins with a nuclear RNase III Drosha together with the cofactor DGCR8 that cut the stem–loop to release a pre-miRNA [6,7]. Maturation is completed in the cytoplasm where the pre-miRNA is exported through the complex EXP5–Ran·GTP [8]. Here, pre-miRNA is cleaved by Dicer, a RNase III family protein, generating a small RNA duplex ~22 nucleotides in length. Many studies describe the involvement of Drosha and Dicer alterations in cancer [9,10]. Karube and colleagues have demonstrated that the downregulation of Dicer expression in lung cancers is associated with lower postoperative survival [9]. Another study by Merritt and colleagues suggests that alteration in miRNA processing, due to low levels of Dicer and Drosha proteins, could lead to a poor clinical outcome of ovarian cancer patients [10]. 

The pre-miRNA is subsequently loaded on Ago protein to form a complex called pre-RNA-induced silencing complex (pre-RISC), that removes the passenger strand to generate a mature RISC [11]. The mature complex is necessary for miRNA binding to its target mRNA, this leads to the silencing of the mRNA through mRNA cleavage or translational repression or deadenylation, depending on miRNA affinity to the mRNA target [11]. A single miRNA can bind to a large spectrum of different mRNAs, suggesting that miRNAs have a key regulatory role in many biological processes. Indeed, deregulation in miRNA expression is implicated in different diseases, including cancer. In particular, miRNAs can act at different stages of malignant transformation such as initiation, malignant conversion, progression and metastasis [12]. 

At the genomic level, many miRNA sequences are located in intergenic regions, others are encoded by introns of coding transcripts together with their host genes. Some miRNAs are close to each other and are regulated by the same promoter, constituting a polycistronic sequence containing multiple loops from which mature miRNAs are processed [4,11]. An example of this is the miRNA-17/92 cluster (miR-17/92), known as “oncomiR-1”, which is highly conserved among vertebrates [13]. Several studies reported that this cluster is often deregulated in hematopoietic and solid cancers such as colorectal cancer, head and neck cancers, pancreatic cancer, breast cancer, ovarian cancer, lung cancer, renal cancer, hepatocellular carcinoma and osteosarcoma [14]. Many miRNAs are located in cancer-associated genomic regions or in fragile sites, playing an important role in human cancer as oncogenes or tumor suppressor genes [15]. Oncogenes are those miRNAs whose expression is upregulated in tumors. These oncogenic miRNAs, also known as “oncomirs”, promote cancer progression by inhibiting the expression of tumor suppressor genes involved in different biological processes [16].

The development of different high-throughput miRNA profiling technologies, such as miRNA microarray and RNA sequencing, has allowed the characterization of miRNA expression profiles for several malignancies [17]. Many studies reported miRNA-expression profiles of human tumors to identify miRNAs associated with diagnosis, staging, progression, prognosis and response to treatment [18]. Recently, Lu and colleagues have identified at least 10 miRNAs significantly associated with oral cancer [19]. In particular, miR-10b actively participates in cancer formation by promoting cell migration and invasion [19]. miR-17-92 cluster has been characterized to be overexpressed in osteosarcoma, determining a deregulation of several genes involved in differentiation (RGMB, LRRC17), cell cycle control (CCNE1), and apoptosis (LIMA1, CAMK2N1) [20]. Kim and colleagues have identified a series of miRNAs (miR-20a, miR-25, miR-93, miR-103, miR-106a, miR-106b, miR-130, miR-155, miR-221 and miR-222) associated with tumor penetration through serosa, lymphonode metastasis and distant metastasis in gastric cancer [21]. miR-21-3p, miR-96-5p, miR-141-3p and miR-130b-3p have been described to be deregulated in oral squamous cell carcinoma [22]. In particular, the concomitant inhibition of these four miRNAs leads to the decrease of cell proliferation and to the reduction of Cyclin D1 protein levels [22]. 

In this review, we will describe recently published evidence on the involvement of oncogenic miRNAs in several common human cancers through the regulation of different crucial cellular pathways. Finally, we will focus our attention on the potential application of miRNAs as biomarkers, diagnosis and therapeutic tools for human cancers.

## 2. Oncogenic MiRNAs in the Control of Cell Proliferation

Alteration in cell cycle is often considered a principal cause of cancer occurrence [23]. In an adult organism, most of the cells are quiescent and only specialized cells, which are present in hematopoietic system or in the gut epithelium, maintain active proliferation. There are many mechanisms that control these important statuses and the occurrence of imbalances in these regulatory pathways can lead to cancer development. 

Regulation of cell cycle, including detection and repair of genetic damages, is controlled by a series of checkpoints [24]. The main regulatory proteins of cell cycle, cyclins and cyclin-dependent kinases (CDKs), determine cell progression through the cell cycle [25]. 

**Figure 1:**
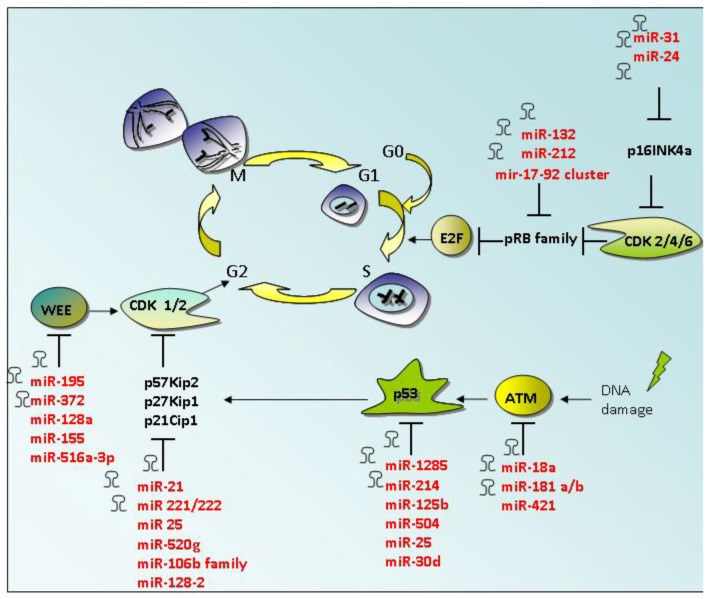
Oncogenic miRNAs involved in cell cycle progression. Cell cycle is divided into four phases, G1, S, G2, and M. Regulation of the cell cycle, including the detection and repair of genetic damage, is controlled by a series of checkpoints. Cyclins and cyclin-dependent kinases (CDKs) are key proteins that determine cell progression through the different phases of cell cycle. Oncogenic miRNAs contribute to cell cycle entry and progression by targeting CDK inhibitors or tumor suppressor genes involved in cell cycle. ATM (Ataxia Telangiectasia Mutated).

Oncogenic miRNAs that have an impact on cell cycle, determining an increase in cell proliferation, contribute to cell growth by targeting CDK inhibitors or transcriptional repressors of the retinoblastoma family proteins (Table 1 and Figure 1). A large-scale analysis of miRNA profiles of solid tumors showed an up-regulation of the human miR-17-92 cluster in many cancers, including lung cancer [26]. This cluster is composed of seven miRNAs and it is located in intron 3 of the C13orf25 gene. The study of Lu and colleagues identified the tumor suppressor Rbl2 as a new target of mir-17-92 cluster, suggesting that the cluster promoted the highly proliferative and undifferentiated phenotype of lung epithelial progenitor cells [27]. In a recent study by Motoyama and colleagues, mir-17-92 cluster has been identified to be over-expressed also in colorectal cancer tissues compared to normal epithelial tissues by miRNA array analysis [28]. Park and colleagues reported that miR-132 and miR-212, that resulted to be over-expressed in pancreatic adenocarcinoma tissues, promote cell proliferation by targeting the retinoblastoma tumor suppressor, Rb1 [29].

**Table 1:** Oncogenic miRNAs involved in cell cycle progression.

Cell Cycle Regulator	Oncogenic miRNAs	Cancer Type	Reference
Rbl2	miR-17-92 cluster	Lung cancer	[27]
Rb1	miR-132, miR-212	Pancreatic adenocarcinoma	[29]
Wee1	miR-195, miR-128a, miR-155, miR-516a-3p, miR-372	Melanoma, Pituitary adenomas	[30,31]
p53	miR-504, miR-25, miR-30d, miR-125b, miR-1285, miR-214	Osteosarcoma, Lung cancer, Neuroblastoma, Colorectal carcinoma, Ovarian cancer, Hepatoblastoma, Breast cancer	[32,33,34,35,36]
p16INK4a	miR-24, miR-31	Human diploid fibroblasts and Cervical carcinoma, mouse embryonic fibroblasts	[37,38]
p57Kip2	miR-21, miR 221/222 cluster, miR-25	Prostate cancer, Gastric cancer	[39,40]
p27Kip1	miR-221, miR-222	Glioblastoma, Chronic lymphocytic leukemia, Breast cancer, Gastric cancer	[40,41,42,43]
p21	miR-520g, miR-106b family, miR-128-2	Non-small-cell lung cancer, Colorectal Cancer, Breast cancer, Kidney cancer, Gastric cancer, Ductal carcinoma of the breast, Barrett's esophagus, Prostate cancer	[44,45,46,47]
ATM	miR-18a, miR-181 a/b, miR-421	Colorectal cancer, Breast cancer, Neuroblastoma	[48,49,50]

This considerable amount of evidence highlights an involvement of oncogenic miRNAs in the control of cell cycle entry and G1/S transition. The regulatory role of miRNAs subsists also in later phases of the mammalian cell cycle [51]. Upon DNA damage cells can be arrested at G2/M phase of cell cycle. The G2/M transition is activated by Cdc25-mediated dephosphorylation of the cyclin B/CDK1 complex [52]. Wee1, a nuclear serine/threonine kinase protein, is a negative regulator of G2/M checkpoint that acts by phosphorylating cyclin B/CDK1 at position Tyr15, determining protein complex inactivation [51]. Bhattacharya and collaborators, by analyzing the expression levels of Wee1 in a series of melanoma patient samples and melanoma cell lines, found an inverse correlation between the expression of Wee1 and miR-195 [30]. MiR-195 regulation of Wee1 expression in malignant melanoma overcame cell cycle arrest upon stress conditions and an unrestricted growth of tumor cells [30]. Wee1 expression has been shown to be regulated by miR-128a, miR-155 and miR-516a-3p, in pituitary adenomas [31]. In regards to the role of miRNAs in maintaining normal stem cell division and renewal, Qi *et al.* demonstrated that in human embryonic stem cell depleted for Dicer and Drosha expression, normal cell growth can be partially restored by the introduction of the mature miR-195 and miR-372 that act by regulating Wee1 and p21 protein expression [53]. p21 protein is a transcriptional target of p53 which mediates growth arrest when cells are exposed to DNA damaging agents such as doxorubicin and ionizing radiation [54]. p21 protein acts by inhibiting the activity of cyclin/cdk2 complexes, and determining the inhibition of cell cycle progression [55]. Zhang and colleagues have identified a novel p53/miR-520g/p21 signaling axis that regulates the response of colon cancer cells to chemotherapeutic agents [44]. In particular, miR-520g confers drug resistance by the inhibition of p21 expression [44]. MiR-106b family exerts anti-apoptotic and cell cycle-promoting effects *in vitro* and tumorigenic activity *in vivo* by promoting the G1-to-S cell cycle transition through p21 silencing [45]. Different lines of evidence demonstrate that miR-106b is also involved in esophageal neoplastic progression and proliferation and that it is also able to confer resistance to irradiation in prostate cancer [46,47]. 

### 2.1. Modulation of p53 Protein by MiRNAs

The p53 tumor-suppressor protein exerts anti-proliferative effects, as well as growth arrest and apoptosis, in response to different intrinsic and extrinsic stress signals including loss of fidelity in DNA replication, genomic instability, DNA damage, unfaithful chromosome segregation and improper mitogenic stimulation [56]. p53 protein acts as a transcriptional activator and regulates the expression of many target genes involved in different cellular processes, such as cell cycle arrest, apoptosis, DNA repair and senescence [57]. Various studies have demonstrated that oncogenic miRNAs are implicated in the regulation of p53 expression [32,33,34,35,36]. Particularly, in a recent study by Hu and colleagues, it has been demonstrated that miR-504 acts as negative regulator of p53 through its direct binding to two sites of p53 promoter [32]. They demonstrated that miR-504 reduces p53-mediated apoptosis in U2OS osteosarcoma cells and H460 lung cancer cells and that it reduces p53-mediated cell cycle arrest in colon cancer [32]. Moreover, miR-504 promotes tumorigenesis through its negative regulation of p53 protein levels *in vivo* [32]. Kumar and colleagues have identify miR-25 and miR-30d, as miRNAs targeting the 3′UTR of TP53, thus showing their capability in adversely affecting apoptotic cell death, cell cycle arrest and cellular senescence in colon cancer [33]. Consequently, depletion of either miR-25 or miR-30d expression, increases endogenous p53 protein expression levels and cellular apoptosis in different cancer cell lines [33]. miR-125b, a brain-enriched miRNA, has been identified as negative regulator of p53 protein in both zebrafish and humans [34]. Indeed, the over-expression of miR-125b determines the repression of the endogenous p53 protein and consequent inhibition of apoptosis in human neuroblastoma cells and human lung fibroblasts [34]. miR-1285 has been demonstrated to regulate the expression of p53 by targeting its 3′UTR and consequently to suppress the expression of p21 [35]. MiR-214 has been demonstrated by Xu and collogues to have an oncogenic role in ovarian cancer stem cells by targeting p53 and determining Nanog induction and chemoresistance [36]. 

These results suggest that some miRNAs exert their oncogenic activity by negatively regulating human TP53 gene expression, adversely regulating apoptosis, cell cycle arrest and senescence of cancer cells, thus they could be considered attractive targets for new drug therapies.

Mutations in the TP53 gene are the most frequent type of gene-specific alterations in different human cancers [58]. Most of these mutations (90%) are missense mutations that mainly reside in the exons encoding the p53 DNA-binding domain [59]. These mutations frequently cause a loss of wild-type p53 tumor suppressor activity, but at the same time, some of these mutant p53 proteins gain new oncogenic properties that favor insurgence, maintenance, spread of the tumor, and chemoresistance of malignant cells [60,61]. Up until now two different molecular mechanisms through which mutant p53 exerts its gain of function activity have been characterized: (a) even if mutant p53 is not able to bind to DNA, it can be recruited by different transcription factors on the promoter of its gene targets, different from those commonly recruited by wild type p53 protein; (b) otherwise mutant p53 can bind to and sequester different tumor suppressor proteins, such as its family members p63 and p73 [62,63,64,65]. Recently, Donzelli and colleagues have demonstrated that mutant p53 protein is also able to modulate the expression of miRNA genes [66]. In particular, they showed that mutant p53 can be recruited on the promoter of miR-128-2 host gene, ARPP-21, inducing the expression of miR-128-2, that in part contributes to mutant-p53-mediated chemoresistance to different drugs [66]. Ganci and colleagues also reported the involvement of mutant p53 in the control of miRNAs expression [67]. They performed miRNA expression profiling in a casuistry of head and neck squamous cancer patients, analyzing also p53 protein status [67]. In particular, they identified 12 miRNAs whose expressions were correlated with short recurrence-free survival, and a group of four miRNAs that correlated with cancer-specific survival, which also associated with p53 mutations [67]. 

Neilsen and colleagues showed that miR-155 is up-regulated by mutant p53 in breast tumors and that it contributes to mutant p53 gain of function activity by inhibiting ZNF652 protein expression, a novel zinc-finger transcriptional repressor [68]. ZNF652 directly repressed key drivers of invasion and metastasis, such as TGFB1, TGFB2, TGFBR2, EGFR, SMAD2 and VIM [68]. 

This set of evidence highlight an important role also for mutant p53 in the modulation of oncogenic miRNAs expression, through which it exerts its gain of function activity.

### 2.2. Oncogenic MiRNAs Regulated by MYC and RAS

The proto-oncogene MYC encodes a transcription factor that regulates cell proliferation, growth and apoptosis [69]. Deregulation of MYC is one of the most common abnormalities in human malignancy [70]. MYC directly regulates the expression of a different set of miRNAs that contribute to tumorigenesis [71,72,73,74]. Schulte and colleagues have identified *in vitro* seven miRNAs (miR-92, miR-106a, let-7b, miR-17-5p, miR-93, miR-99 and miR-221) induced by MYCN, a member of the MYC family, and positively correlating with MYCN-amplification in neuroblastoma [71]. O'Donnell *et al.* reported that miR-17-5p, miR-92 and miR-106a were upregulated by c-MYC in B-cells, suggesting that c-MYC regulates the miR-17 and miR-106a clusters [72]. MYC and MYCN activated miR-9, binding directly to the mir-9 locus in neuroblastoma cells [73]. Mestdagh *et al.* have identified a miRNA signature for MYCN/c-MYC signaling in neuroblastoma tumors [74]. They have shown binding of MYCN to the promoter of several miRNAs, suggesting a direct role for MYCN in the regulation of these miRNAs [74]. RAS also plays a critical role in cell growth control by initiating multiple mitogenic signal transduction pathways and its co-expression with MYC is responsible of cellular transformation [75,76]. Recently, Wang *et al.* have discovered that miR-155 is upregulated after activation of K-Ras in a doxycyline-inducible system [77]. MiR-155 caused inhibition of Foxo3a, which lead to decrease in SOD2 and catalase antioxidants, and enhanced pancreatic cell proliferation induced by ROS [77]. It has been demonstrated that miR-21 is upregulated both *in vitro* and *in vivo* by Ras [78]. In particular, miR-21 is upregulated in thyroid tumors with anaplastic histotype, and in mice miR-21 knockdown is able to repress the growth of tumors xenografts [78]. Moreover, in mice lung tumors, miR-21 is induced by oncogenic Ras activation [78]. In this study, the authors also showed a correlation between miR-21 levels and early stages of non-small-cell lung cancers [78]. A genome-wide screening for miR-21 targets, finally, showed that the miR-21 overexpression deregulates a network of genes involved in cell cycle checkpoints regulators, suggesting a significant role for miR-21 in oncogenic Ras-induced cell proliferation [78].

## 3. Oncogenic MiRNAs in the DNA Damage Response Pathway

Normal cells are able to arrest or delay cell cycle progression in the presence of DNA damage, defective replication or mitotic aberrancies. DNA damage response is the first reaction to genotoxic damage. Alterations in the components of the DNA repair machinery is also associated with tumorigenesis and chemoresistance [79]. DNA damage response requires either ATM or ATR proteins that have the ability to bind to damaged DNA and to activate p53 tumor suppressor protein that in turn leads to DNA repair, cell-cycle arrest or apoptosis, depending on the entity of the damage [80]. ATM and ATR proteins also trigger a phosphorylation cascade that activates two kinases Chk1 and Chk2 [81]. These two kinases stimulate the expression of DNA repair enzymes known as CDK inhibitors (CKIs). There are two families of CKIs based on their structures and their CDK targets. The first class includes the INK4 proteins (inhibitors of CDK4 and CDK6): p16INK4a, p15INK4b, p18INK4c and p19INK4d [82]. The second one includes p21, p27Kip1 and p57Kip2 protein that act as inhibitors of the Cip/Kip family whose actions affect the activities of cyclin-D, E, and A dependent kinases [82]. 

It has been demonstrated that different oncogenic miRNAs have cell cycle inhibitors of the INK4 or Cip/Kip families as targets, determining an aberrant DNA damage response [37,38,39,40,41,42,43]. Lal *et al.* demonstrated that the miR-24 suppresses p16INK4a expression in human diploid fibroblasts and cervical carcinoma cells [37]. Malhas and colleagues demonstrated that miR-31 binds to p16Ink4a/p19Arf 3′UTR determining its inhibition and contributing to cell cycle progression [38]. Inactivation of p57Kip2 protein is commonly observed in cancers and where it was regulated by miR-21 in prostate cancer and by miR-221/222 cluster and miR-25 in gastric cancer [39,40]. Several studies reported that also p27Kip1 protein expression is modulated by miRNA [40,41,42,43]. Two examples of this are miR-221 and miR-222, which have been demonstrated to inhibit p27Kip1 protein expression in different cancer types, including glioblastoma, chronic lymphocytic leukemia, breast cancer and gastric cancer, determining the promotion of cancer cells growth [40,41,42,43]. 

Recent studies have reported ATM protein regulation by miRNAs [48,49,50]. Wu and colleagues demonstrated that miR-18a binds to ATM 3’UTR determining its depletion in colorectal cancer [48]. Bisso *et al.* have demonstrated that ATM is down-regulated by miR-181a/b in triple negative breast cancer [49]. It has also been shown that N-MYC, an oncogene that is often highly expressed in human neuroblastoma cells, inhibited the expression of ATM through miR-421 regulation [50].

These several lines of evidence show miRNA’s ability to control cell-cycle progression at different levels by targeting the proteins involved in the response to cellular damage. 

## 4. Apoptotic Pathway and Oncogenic MiRNAs

Activation of apoptotic pathway plays a key role in biological development and tissues homeostasis [83]. Defects in apoptotic pathway contribute to the occurrence of different human diseases, such as cancer [84]. In particular, alterations in the apoptotic response in cancer can promote tumor initiation, progression and treatment resistance [85]. Several different studies have demonstrated that some oncogenic miRNAs are involved in the control of apoptosis and exert their anti-apoptotic effects by directly targeting pro-apoptotic mRNAs [86]. Apoptosis may occur via two major pathways: the extrinsic and intrinsic pathways. The extrinsic pathway requires the activation of death receptors anchored at the cell membrane through the binding of FasL, TNF and TRAIL ligands, which cause the recruitment and the oligomerization of Fas-Associated protein with Death Domain (FADD) within the Death-Inducing Signaling Complex (DISC), resulting in Caspase-8 and Caspase-10 activation [87]. The intrinsic pathway occurs in the mitochondria and acts as key regulator of apoptosis. Upon DNA damage or cellular stress, p53 protein regulates the cellular destiny by inducing apoptosis and activating the transcription of several pro-apoptotic BCL-2 family members. This results in the release of several mitochondrial proteins, including Cytochrome c (Cyt c), in the cytosol. Cyt c binds to APAF-1 and Pro-Caspase-9, forming a Caspase-9 activating complex or Apoptosome. Caspase-9 cleaves and thereby activates executioner Caspase-3 [88]. The BCL-2 protein family includes anti-apoptotic proteins such as BCL-2, BCL-XL, MCL1, A1, BFL-1, and pro-apoptotic proteins such as BAD, BIK, BID, BIM, PUMA, NOXA, BMF, BAX and BAK [89]. 

After the activation of Apoptosoma, BAX and BAK proteins lead to an increase in the permeability of the mitochondrial outer membrane, that in turn determines the translocation of mitochondrial proteins into the cytosol. BCL-2 and BCL-XL exert their anti-apoptotic function by sequestering BAX and BAK and thereby decreasing the mitochondrial membrane permeability [90]. Caspase activation is a crucial event in cellular death and it is responsible of the cleavage of several critical cellular substrates determining cellular destruction [91].

**Figure 2:**
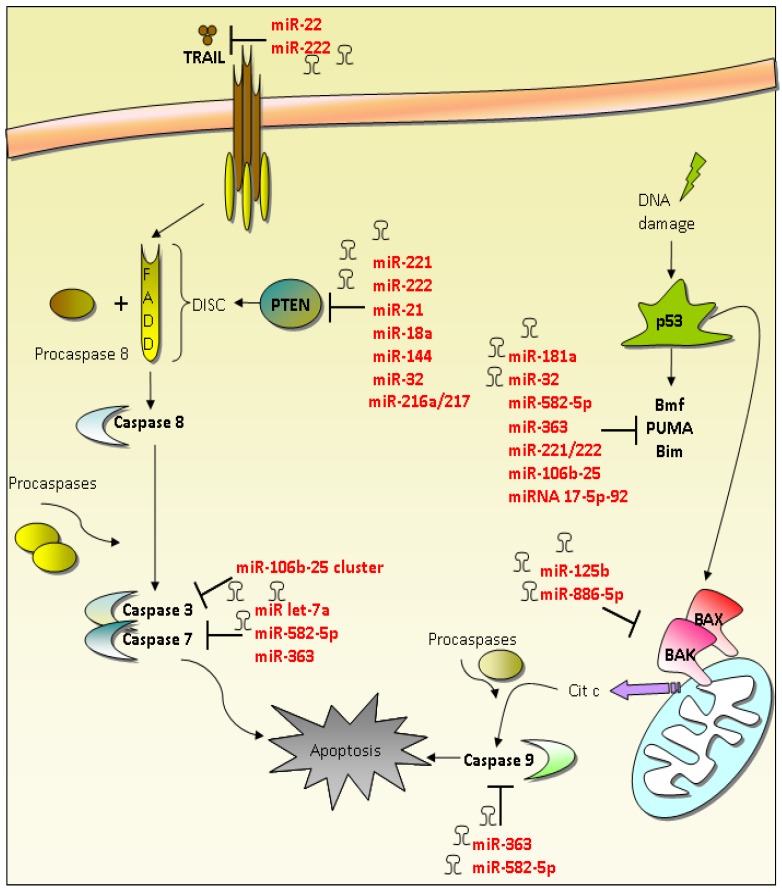
Oncogenic miRNAs involved in the apoptotic pathway. Anti-apoptotic miRNAs exert their function in both extrinsic and intrinsic apoptotic pathways by regulating pro-apoptotic mRNAs including caspases. Caspase 3-7-9 are downregulated by miR-106b-25 cluster, miR let-7, miR-582-5p and miR-363. TRAIL ligand, involved in extrinsic pathway is downregulated by miR-22 and miR-222. Also PTEN, that promotes the formation of the Death-Inducing Signaling Complex Apoptosis, is regulated by several miRs. BCL-2 family members such as PUMA, BMF, BAX and BAK members, involved in intrinsic pathway, are downregulated by many anti-apoptotic miRNAs, which lead to resistance to apoptosis. FADD (Fas-Associated protein with Death Domain); TRAIL (TNF-Related Apoptosis-Inducing Ligand); DISC (Death-Inducing Signaling Complex); PTEN (Phosphatase and Tensin homolog); BMF (Bcl2 Modifying Factor); PUMA (BCL2 binding component 3); BIM (BCL2-like 11); BAX (BCL2-Associated X protein); BAK (BCL2-Antagonist/Killer 1).

Anti-apoptotic miRNAs can exert their functions at different levels in both extrinsic and intrinsic pathways by regulating pro-apoptotic mRNAs (Table 2 and Figure 2).

**Table 2:** Oncogenic miRNAs involved in apoptotic pathway.

Pro-Apoptotic Target	Oncogenic miRNAs	Cancer Type	Reference
TRAIL	miR-221, miR-222	Non-small-cell lung cancer	[92]
PTEN	miR-221, miR-222, miR-21, miR-18a, miR-144, miR-32, 216a/217	Non-small-cell lung cancer, Hepatocellular carcinoma, Gastric cancer, Nasopharyngeal carcinoma, Colorectal carcinoma, Liver cancer	[92,93,94,95,96,97,98,99]
Bax	miR-886-5p	Cervical cancer	[100]
Bak	miR-125b	Prostate cancer, Breast cancer	[101,102]
Bmf	miR-221	Hepatocellular carcinoma	[103]
PUMA	miR-221/222	Epithelial cancers	[104]
Bim	miR-181a, miR-17-5p-92 cluster, miR-32, miR-106b-25 polycistron, miR-582-5p, miR-363	Non-Hodgkin lymphoma, Neuroblastoma, Prostate cancer, Esophageal cancer, Glioblastoma	[105,106,107,108]
Caspase-7	miR-106b-25 cluster	Prostate cancer	[109]
Caspase-3	miR-582-5p and miR-363, miR let-7a	Glioblastoma, Squamous carcinoma	[108,110]
Caspase-9	miR-582-5p and miR-363	Glioblastoma	[108]

### 4.1. Regulation of Extrinsic Apoptosis Pathway by MiRNAs

Acunzo *et al.* demonstrated that miR-130a is able to reduce drug resistance in non-small-cell lung cancer by targeting MET protein and inhibiting its activation of the oncogenic miR-221 and miR-222 [92]. The oncogenic miR-221 and miR-222 increase cell survival and induce TRAIL resistance through the downregulation of p27kip1, PTEN and TIMP3 proteins [92]. PTEN is one of the most frequently mutated tumor suppressors in human cancer and is implicated in the apoptotic pathway through the promotion of the DISC complex formation [111]. Several studies reported a miRNA-dependent regulation of PTEN protein [93,94,95,96,97,98,99]. An example of this is miR-21 that negatively regulates PTEN protein in many tumors such as gastric cancer, non-small-cell lung cancer and hepatocellular carcinoma [93,94,95]. PTEN protein has also been demonstrated to be a target of miR-18a, miR-144, miR-32 and miR-216a/217 [96,97,98,99].

### 4.2. Regulation of Intrinsic Apoptosis Pathway by MiRNAs

In regards to the oncogenic miRNA-mediated regulation of the intrinsic apoptotic pathway, miR-886-5p is an example. It has been demonstrated to be up-regulated in cervical cancer and reduce apoptosis by directly down-regulating BAX protein expression [100]. Also miR-125b that targets BAK1 and which is highly expressed in prostate cancer and in breast cancer cells, contributes to disease progression and chemoresistance [101,102]. MiR-221 and miR-222, that are frequently up-regulated in human epithelial cancers and whose role as oncogene has been deeply characterized, have been demonstrated to be also implicated in the regulation of the apoptotic intrinsic pathway [103]. In particular, Gramantieri and colleagues, reported that miR-221 inhibits apoptosis in hepatocellular carcinoma by targeting BMF protein and that its over-expression was associated with a more aggressive phenotype [103]. Also PUMA has been demonstrated to be a direct target of miR-221/222 in epithelial cancers [104]. Lwin and colleagues have demonstrated that miR-181a confers drug resistance of non-Hodgkin lymphoma by targeting BIM protein [105]. In neuroblastoma, miR-17-5p-92 cluster have been shown to inhibit p21 and BIM protein translation by interacting with their 3′UTRs [106]. Moreover, miR-17-5p depletion has been demonstrated to abolish tumor growth *in vivo* [106]. Ambs and colleagues reported higher levels of miR-32 in primary prostate tumors compared to prostate tissues. They also demonstrated that BIM protein is a target of miR-32 [107]. Kan and colleagues demonstrated that miR-106b-25 polycistron is activated by genomic amplification and is potentially involved in esophageal neoplastic progression and proliferation via suppression of BIM protein expression [46].

### 4.3. Caspases and Anti-Apoptotic MiRNAs

Many lines of evidence demonstrate that miRNAs can control apoptosis through the regulation of caspase expression [108,109,110]. MiR-106b-25 cluster, that has been shown to be up-regulated in human prostate cancer, acts in part by the inhibition of caspase-7 expression [109]. In addition, high levels of miR-106b have been shown to be associated with disease recurrence [109]. The combination of high miR-106b and low caspase-7 expression levels, in primary tumors is an independent predictor of early disease recurrence [109]. MiR-106b-25 cluster influences also focal adhesion-related pathways [109]. In particular, Hudson and colleagues demonstrated that after infection with a miR-106b-25 lentiviral expression construct, prostate cancer cells showed an increased adhesion to basement membrane and bone matrix-related filaments [109]. In a recent study, Floyd and colleagues demonstrated that miR-582-5p and miR-363 inhibit apoptosis by directly targeting Caspase-3, Caspase-9, and BIM proteins in glioblastoma [108]. Caspase-3 has been shown to be a target of miR let-7a in human squamous carcinoma cells and hepatocellular carcinoma cells [110].

All this evidence supports a notable role for miRNAs as key regulators of apoptotic players. Understanding the regulatory mechanisms and the signaling pathways that control apoptosis can have an important impact on the development of novel therapeutic strategies for cancer, especially for those types of tumors that have a decreased sensitivity to treatments.

## 5. Oncogenic MiRNAs as Pro-Metastamir 

Metastasis is one of the principal causes of death in cancer patients [112]. Metastasization involves multiple steps that allow cells to access body cavities, or circulatory systems, their survival during transport until they arrest at discontiguous sites, the exit from circulation, and their proliferation at ectopic sites in response to local growth factors [113]. More recently, a specific family of miRNAs, which are called metastamir, has been shown to have pro- and anti-metastatic effects [114]. Several studies have demonstrated positive or negative feedback loops between upstream effectors of metastatic process and miRNAs [115,116,117]. For example, miR-21 has a role not only in tumor growth, but also in invasion and tumor metastasis by targeting multiple tumor/metastasis suppressor genes such as TPM1, PDCD4 and Maspin in metastatic breast cancer [118]. Asangani and colleagues have demonstrated that miR-21 binds to Pdcd4 3’UTR determining its downregulation and consequentially an induction of invasion processes in colorectal cancer [119]. More recently, Yin *et al.* have identified a signature of upregulated miRNAs (miR-126, miR-141 and miR-21) in the serum that is significantly correlated with the presence of early stage colorectal liver metastasis [120]. MiR-10b is overexpressed in metastatic breast cancer cells and it positively regulates cell migration and invasion. In particular, the expression of miR-10b is induced by the transcription factor Twist, which binds to the putative promoter of mir-10b [121]. Liu and colleagues have observed that, in gastric cancer, miR-10b levels are upregulated in lymphoma node metastasis-positive tumor tissues compared with lymphoma node metastasis-free tumor tissues [122]. In gastric tumors, miR-10b determines the upregulation of RhoC and the induction of AKT phosphorylation through targeting HOXD10, thus promoting cell invasion [122]. A recent study proposed the miR-10b/HOXD10/MMP-14/uPAR signaling pathway as responsible of the invasion of glioma [123]. In particular, miR-10b modulates invasion factors MMP-14 and uPAR expression via the target HOXD10 gene [123]. Ma and colleagues have identified miR-9 as a pro-metastatic miRNA and a negative regulator of the key metastasis suppressor E-cadherin, in breast cancer [73]. It has been demonstrated that miR-373 and miR-520c promote migration in non-metastatic MCF7 human breast cancer cells and that *in vivo* they enhance metastasis at least in part by targeting the adhesion molecule CD44 [124]. In a recent study, miRNA profiles, generated by analyzing a casuistry of primary colorectal cancers (pCRCs) and matched liver metastases (LMs), reported miR-885-5p to be significantly upregulated in LM compared with pCRC tissues, and high serum levels of miR-885-5p to be significantly predictive of CRC prognosis and metastasis [125]. These results highlight the potential role of serum miR-885-5p as a noninvasive CRC biomarker [125]. 

All of these studies provide a rationale for further exploring these newly discovered miRNAs as metastasis biomarkers. 

## 6. Oncogenic MiRNAs and Drug Resistance 

Drug resistance is a major factor that limits the success of chemotherapy. Tumors can be intrinsically resistant before chemotherapy treatment, or resistance may be acquired during therapy [126]. In both cases the effect is an inefficient intervention that results in a worsening of the disease. Recent studies have demonstrated that oncogenic miRNAs are responsible for drug resistance by interfering with DNA-repair pathway and apoptotic pathway. For example, miR-125b plays a role in the increase of chemoresistance to doxorubicin, vincristin, etoposide and mafosmamide in Ewing sarcoma/primitive neuroectodermal tumor (EWS), by suppressing the expression of apoptotic mediators, such as p53 and Bak proteins [127]. Bak is also downregulated by miR-125b in prostate cancer and breast cancer, contributing to disease progression and resistance to paclitaxel treatment [101,102]. Valeri and colleagues have demonstrated that in colorectal cancer, miR-21 targets and down-regulates human mutS homolog 2 (hMSH2) and 6 (hMSH6), the core mismatch repair (MMR) recognition protein complex, that in turn determines an increased resistance to 5-fluorouracil treatment [128]. MiR-128-2 determined an increase in chemoresistance to different anticancer drugs (cisplatin, doxorubicin and 5-fluorouracil) in lung cancer cells [66]. 

As described previously, PTEN plays a role in the apoptotic pathway and its inactivation often determines drug resistance. Liu and colleagues demonstrated that miR-21 binds to PTEN 3’UTR, determining its depletion and inducing chemoresistance in non-small-cell lung cancer [93]. MiR-21 has been also identified to confer chemoresistance to gemcitabine in malignant human cholangiocytes and to cisplatin in gastric cancer, through the modulation of PTEN and AKT proteins [129,130]. MiR-17-5p induces drug resistance in ovarian cancer cells by the modulation of PTEN signaling [131]. 

The alteration of the caspase activity may be a cause of drug resistance too. Zhang and colleagues have demonstrated that miR-217 induces the resistance of breast cancer cells to the effects of chemotherapy reagents by inhibiting the caspase 3/7 activities [132]. It has also been demonstrated that let-7a increases resistance to interferon-gamma, doxorubicin and paclitaxel in human squamous carcinoma and hepatocellular carcinoma cells by targeting caspase-3 [110]. 

All these lines of evidence highlight an important role of oncogenic miRNAs in cancer cell treatment response, suggesting a possible use of miRNAs as a target of new therapy approaches in combination with the common anticancer drugs.

## 7. Conclusions and Future Prospective

Alteration in the expression of oncogenes, tumor-suppressor genes and miRNAs is one of the principal causes that determine cancer occurrence. 

All the knowledge gathered and accumulated over the last several years have shown the importance of miRNAs in many biological processes implicated in cancer, such as cell cycle regulation, apoptosis and differentiation. Several studies have identified miRNA expression profiles specific to different human cancer types that also correlate with patient survival, suggesting the potential usefulness of miRNAs in cancer prediction, diagnosis and therapy [133,134,135]. In a tumoral context, miRNAs can act as oncogene or tumor suppressors in accordance with their mRNA targets and the same miRNAs can exert an opposite function depending on the cellular context. Thus, this makes understanding the molecular mechanisms in which miRNAs are involved in even more difficult.

As previously described, oncogenic miRNAs can promote tumor progression through the inhibition of tumor suppressor proteins involved in the main cellular pathways, which range from cell cycle control to DNA damage response, apoptosis, chemoresistance and migration.

Due to the great number of potential targets for each miRNA there is still so much to be understood regarding the molecular mechanisms of the involvement of each oncogenic miRNA.

The advent of “omics” sciences, such as transcriptomic and proteomic, combined with miRNA expression profiling, has certainly allowed the integration of multiple information sources for a better understanding of the intricate pathways involved in cancer progression. 

Despite this, there are still many open questions regarding miRNA itself in terms of regulation, and miRNA-mediated mRNAs regulation.

Emerging over the last few years is the potential role of miRNAs as powerful biomarkers for cancer screening, diagnosis, prognosis, and therapeutics, due to their presence in the majority of body fluids. Moreover, much effort is in progress in an attempt to generate miRNA targeting therapies to restore the normal miRNAs expression pattern in cancer cells.

### 7.1. MiRNAs as Biomarkers

One of the main challenges in the field of cancer research is the identification of molecular biomarkers that might make early diagnosis possible. 

The main characteristics that identify a potential biomarker as a good one are: easy accessibility, in order to avoid excessively invasive approaches, highly predictive (sensitivity and specificity) in the diagnosis or prognosis of a specific cancer type and high stability in body fluids or tissues. 

Being deregulated in different cancer types with a tissue-specific expression pattern, miRNAs hold promise as biomarkers. In fact, miRNAs present different features common to potential biomarkers. 

MiRNAs have been demonstrated to be easily detectable by minimally invasive methods. Different studies have reported the presence of miRNAs in a variety of body fluids, such as serum, plasma, saliva and urine, as they can be passively released by tumor cells that undergo lysis or can be transferred into exosomes released by the cell membrane [136]. Circulating miRNAs are present both in patients with cancer and in healthy controls, with a different pattern of expression that allow circulating miRNAs can also be early long term predictor for breast cancer [137]. For example, recently, miR-486-5p and miR-938, detected in the peripheral blood have been demonstrated to discriminate patient with prostate cancer from healthy controls [138]. MiR-505-5p, miR-125b-5p, miR-21-5p, and miR-96-5p resulted to be significantly upregulated in plasma of breast cancer patients [139]. In non-small-cell lung cancer high serum levels of miR-486, miR-30d and miR-125, and low serum levels of miR-1 and miR-499 were associated with poor survival [140,141,142]. It has also been demonstrated that some circulating miRNAs can provide important information about the tumor itself. For example, in different cancer types, miR-21 plasma levels successfully predicted chemotherapy response [143,144,145]. Another important feature of miRNAs is their stability. Being small molecules of RNA, miRNAs are characterized by a higher stability compared to messenger RNAs and, for this reason, they are more easily detectable in tissues and body fluids by high-throughput experimental approaches or by simple and universally applicable assays for quantization, such as RT-qPCR.

Although miRNAs present the majority of potential biomarker features, there are still some limitations for their application, such as: the lack of an endogenous control miRNA that can be used to normalize the quantity of a specific miRNA in body fluids, the little knowledge on miRNAs half-life in body fluids, the difficulty to discriminate tumor specific circulating miRNAs as they may derive from various cells, including normal blood cells as well as by cells associated with the tumor microenvironment, the need to establish the correlation between miRNA expression patterns and different patient variables (age, common health conditions and lifestyles), and the lack of standardized methods for miRNAs isolation and quantification. 

For all these reasons, further investigations are required to make the use of miRNAs as biomarkers possible for cancer diagnosis.

### 7.2. MiRNA Targeting Therapies

In the field of applied research, there are numerous attempts underway to develop miRNA targeting therapies with the aim to recover the normal balance in miRNA expression that is frequently altered in tumoral cells. The advantage in targeting a miRNA rather than a single protein is due to the ability of miRNAs to modulate entire gene programs.

One approach consists of the direct delivery of specific miRNAs to their target tissues, but a major limitation is the many barriers under which these molecules undergo, such as low cellular uptake, immune response, degradation by nucleases, and poor endosomal release. The development of non-viral synthetic miRNAs, in part overcomes these obstacles. Recent studies suggest the possibility to directly inhibit oncogenic miRNA activity using miRNA antagonists that are small antisense oligonucleotides with sequences complementary to endogenous mature miRNA. These are synthetic anti-miRNAs or locked nucleic acids (LNAs) [146,147]. This kind of approach unfortunately remains difficult for many cell types such as muscle and brain. 

Despite much effort in miRNA-based therapies, few miRNA therapies have entered clinical trials. An example of this is the liposomal miR-34 mimic, MRX34, which is the first one that has successfully entered a clinical trial for treatment of metastatic cancer with liver involvement and unresectable primary liver cancer [148]. Another successful case is the LNA-modified-anti-miR-122, RG-101, that has completed phase II of clinical trials for the treatment of hepatitis C-virus (HCV) in liver transplants [149].

Slow progress is due to the general difficulties in overcoming technical limitations regarding this approach such as delivery, stability and avoidance of activating immune responses.

Treating cancer with oncogenic miRNA antagonists will require developing novel formulation strategies as well as increasing current knowledge of the underlying mechanisms of action of the miRNAs.
